# Surgical management of sarcoma in adolescent and young adult patients

**DOI:** 10.1186/s13104-020-05107-0

**Published:** 2020-05-26

**Authors:** Kazuhiko Hashimoto, Shunji Nishimura, Naohiro Oka, Masao Akagi

**Affiliations:** Department of Orthopaedic Surgery, Kindai University Hospital, 377-2 Ohno-Higashi, Osaka-Sayama City, Osaka 589-8511 Japan

**Keywords:** Sarcoma, Surgery, Adolescent and young adult, Prognosis

## Abstract

**Objective:**

To examine the clinical features and outcomes of adolescent and young adult sarcoma patients who underwent surgical management and clarify important factors associated with prognosis. We reviewed 18 young adult sarcoma patients sarcoma patients treated surgically in our hospital. The tumor site, histology, grade, stage, and American Society of Anesthesiologists-Physical Status before surgery, operation time, intraoperative blood loss, complications, surgical margin, local recurrence, metastasis, and outcomes were investigated. The 3-year survival rate was also calculated. We compared survival based on age, grade, and surveyed features of poor outcome cases.

**Results:**

The 3-year survival rate was 61.3%. There was no significant difference in survival based on age, grade, operation time, or intraoperative blood loss. Three of five patients who died of the disease had stage ≥ IV at diagnosis. All patients with R1 surgical margins developed recurrence and all those with an American Society of Anesthesiologists-Physical Status ≥ 2 died. Patients with late-stage sarcomas, R1 tumor margin, or high American Society of Anesthesiologists-Physical Status score had poor prognoses. To achieve a favorable outcome in adolescent and young adult sarcoma patients, early detection and obtaining R0 ≥ surgical margin are essential.

## Introduction

Sarcoma is a rare malignancy developing in non-epithelial tissues such as the fat, muscle, and bone [1]. In Japan, the annual incidences of bone and soft tissue sarcomas are 1 and 3 per 100,000 people per year, respectively [2, 3]. Although the incidence account to only 1–2% that of cancers such as colorectal, stomach, and lung, there are more than 50 histological types of sarcomas, and the malignancy varies [4]. Furthermore, sarcoma has no age prevalence [5]. In recent years, oncologists have focused on the generation of patients classified as adolescents and young adults (AYA), which is defined as a group of 15–39 years old individuals with a high concentration of important life events such as schooling, employment, romance, marriage, and childbirth. Sarcomas occurring in this age range represent a unique spectrum of malignancies and are considered to require special care due to their characteristics [6–8]. In addition, the number of cancer and sarcoma patients in the AYA generation has increased [7, 8]. According to previous reports, the 5-year survival rate of sarcoma is around 60–70% [4]. The treatment strategy for and prognosis of sarcomas have improved over the past few decades [9]. However, there have been no recent improvements in treatment, and the survival rate has plateaued [9]. Additionally, prognosis in AYA sarcoma patients has not improved because AYA sarcoma cases are rare, and there are not enough clinical data [10]. Recently, we described the clinical features and outcomes of AYA sarcomas in our hospital [11]. In the current study, we analyzed the data of sarcomas in AYA patients treated surgically and identified the patients that had poor prognosis to determine optimal surgical treatment approaches.

## Main text

### Patients and methods

We reviewed 18 sarcoma cases (10 in the soft tissue and 8 in the bone) treated surgically in our institute between March 2009 and May 2018. Records of eight male patients and ten female patients aged 15–39 years (mean, 33 years) were reviewed retrospectively. Tumor site, histology, grade, stage, European Society for Medical Oncology Guidelines Performance Status (ECOG-PS), American Society of Anesthesiologists-Physical Status (ASA-PS) before surgery, surgical treatment methods, operation time, intraoperative blood loss, complications, surgical margin, local recurrence, metastasis, and outcomes were investigated. Using previously described methods, surgical margins were classified as R0, R1, or R2 [12]. R1 and R2 margins were those in which the residual tumor was detectable microscopically and macroscopically, respectively. We also surveyed features of poor outcomes of the dead of disease (DOD) cases.

### Statistical analysis

The Statmate 5.01 software package was used to assess the 3-year survival rates. The patients’ 3-year survival rates were calculated using the Kaplan–Meier method and differences were assessed using the log-rank test. P < 0.05 was considered to indicate a statistically significant difference.

This study was approved by the Ethics Committee of Kindai University Hospital (approval no.: 31-153) (Osaka, Japan). All patients also provided written informed consent for participation in this retrospective study.

### Results

#### Patient characteristics

The present retrospective study comprised 18 patients (8 men and 10 women) with sarcoma who underwent surgery in our department (Table 1). Among patients with bone tumors, the tumor site was the femur in three patients, tibia in three, fibula in one, and humerus in one. In terms of histology, six had osteosarcoma and two had chondrosarcoma. Six patients had histological high-grade tumors and two had low-grade tumors. One, three, two, and one patient had stage IB, IIA, IIB, and IVB disease, respectively. Five patients had an ECOG-PS score of 0, two had an ECOG-PS score of 1, and one had an ECOG-PS score of 2. Seven patients had an ASA-PS score of 1, and one had an ASA-PS score of 2.

**Table 1:** Clinical features of patients with bone and soft tissue sarcoma in AYA

Patient no.	Age	Bone or soft tissue	Site	Histology	Grade	ECOG-PS	ASA-PS	Chemotherapy	Treatment	Op. time (min)	Blood loss (mL)	Margin	Local recurrence	Metastasis	Follow-up period	Final outcome
1	17	Bone	Tibia	Osteosarcoma (conventional)	High	1	1	NECO-95J	CT, WR	160	500	R1	+	+	43	DOD
2	36	Bone	Tibia	Osteosarcoma (conventional)	High	0	1	NECO-95J	Curettage	108	10	–	+	–	55	NED
3	39	Bone	Femur	Chondrosarcoma grade I	Low	0	1	–	Curettage	240	860	–	–	–	66	CDF
4	33	Bone	Femur	Chondrosarcoma grade II	Low	0	1	–	Curettage	120	160	–	–	–	75	CDF
5	15	Bone	Femur	Osteosarcoma (osteoblastic)	High	2	1	NECO-95J	CT, WR	215	521	R0	–	–	88	CDF
6	36	Bone	Tibia	Osteosarcoma (chondroblastic)	High	0	1	NECO-95J	CT, WR	245	250	R0	–	–	43	CDF
7	27	Bone	Fibula	Osteosarcoma (conventional)	High	0	1	NECO-95J	CT, WR	500	392	R0	–	–	162	CDF
8	29	Bone	Humerus	Osteosarcoma (conventional)	High	1	2	NECO-95J	CT, WR	295	571	R0	–	+	12	DOD
9	35	Soft tissue	Thigh	Leiomyosarcoma	High	1	3	–	WR	245	418	R0	–	+	45	DOD
10	32	Soft tissue	Buttock	Ewing	High	1	2	VAdCA-IE	CT, WR	140	615	R0	–	+	13	DOD
11	35	Soft tissue	Thigh	Synovial sarcoma	High	0	1	IA × 3 (post surgery)	CT, WR	364	282	R1	+	–	20	NED
12	27	Soft tissue	Abdomen	Synovial sarcoma	High	0	1	IA × 3 (post surgery)	CT, WR	102	10	R0	–	–	40	CDF
13	36	Soft tissue	Upper extremity	Liposarcoma	High	1	1	–	WR	95	10	R0	–	+	54	DOD
14	26	Soft tissue	Thigh	Myxoid liposarcoma	Low	0	1	–	WR	140	935	R0	–	–	144	CDF
15	34	Soft tissue	Thigh	Myxoid liposarcoma	High	0	1	IA 3 (pre-surgery)/2 (Post surgery)	CT, WR	145	138	R1	+	–	111	NED
16	33	Soft tissue	Thigh	Synovial sarcoma	High	0	1	IA 3 (pre-surgery)/2 (post surgery)	CT, WR	237	235	R0	–	–	36	CDF
17	37	Soft tissue	Buttock	Myxoid liposarcoma	High	0	1	IA × 3 (post surgery)	CT, WR	103	196	R0	–	–	8	CDF
18	32	Soft tissue	Groin	Myxoid liposarcoma	High	0	1	IA × 3 (post surgery)	WR, RT	191	50	R0	–	–	10	CDF

*y* years, *F* female, *M* male, *CT* chemotherapy, *WR* wide resection, *NECO* Neoadjuvant Chemotherapy for Osteosarcoma, *IA* ifosfamide and doxorubicin, *Mo* month(s), *DOD* dead of disease, *CDF* continuously disease-free, *ECOG-PS* European Society for Medical Oncology Guidelines Performance Status, *ASA-PS* American Society of Anesthesiologists-Physical Status, *Op. time* operation time, *VAdCA-IE* vincristin, actinomycinD, doxorubicin, cyclophosphamide, ifosfamide, etoposide, *NED* no evidence of disease

Among patients with soft tissue tumors, the tumor site was the thigh in five patients, buttock in two, upper extremity in one, abdomen in one, and groin in one.

In terms of histology, five had liposarcoma, three had synovial sarcoma, one had Ewing sarcoma, and one had leiomyosarcoma. Nine patients had histological high-grade tumors, and one had a low-grade tumor. One, two, three, two, and two patients had stage I, II, IIIA, IIIB, and IVB disease, respectively. Seven patients had an ECOG-PS score of 0, and three patients had an ECOG-PS score of 1. Eight patients had an ASA-PS score of 1, one had an ASA-PS score of 2, and one had an APA-PS score of 3.

#### Treatment

Five patients with bone tumors received wide resection with neoadjuvant chemotherapy (NECO-95J) [13], and three received curettage resection. Of the patients who received wide resection, four achieved R0 resection and one achieved R1 resection. Six patients with soft tissue tumors received wide resection with neoadjuvant chemotherapy (five patients: 5 g/m^2^ ifosfamide and 75 mg/m^2^ doxorubicin [14], and one patient: 1.5 mg/m^2^ vincristine, 37.5 mg/m^2^ doxorubicin, 1200 mg/m^2^ cyclophosphamide, 1.8 g/m^2^ ifosfamide, and 100 mg/m^2^ etoposide for Ewing sarcoma [15]). Four patients received wide resection without any preoperative therapy, although one received postoperative radiation therapy (60 Gy: 2 Gy/day, 5 days/week). Eight of the patients with soft tissue tumors achieved R0 resection and two achieved R1 resection.

The mean operation time for all patients was 175.5 min (range, 95–500 min). The mean operation time for patients with bone tumors was 142.5 min (range, 95–364 min), and the mean operation time for patients with soft tissue tumors was 227.5 min (range, 108–500 min). The mean intraoperative blood loss in all patients was 266 mL (range, 10–935 mL). The mean intraoperative blood loss in patients with bone tumors was 446 mL (range, 10–860 mL), and the mean intraoperative blood loss in patients with soft tissue tumors was 215.5 mL (range: 10–935 mL). There were no postoperative complications in all current cases.

#### Outcomes

**Fig. 1:**
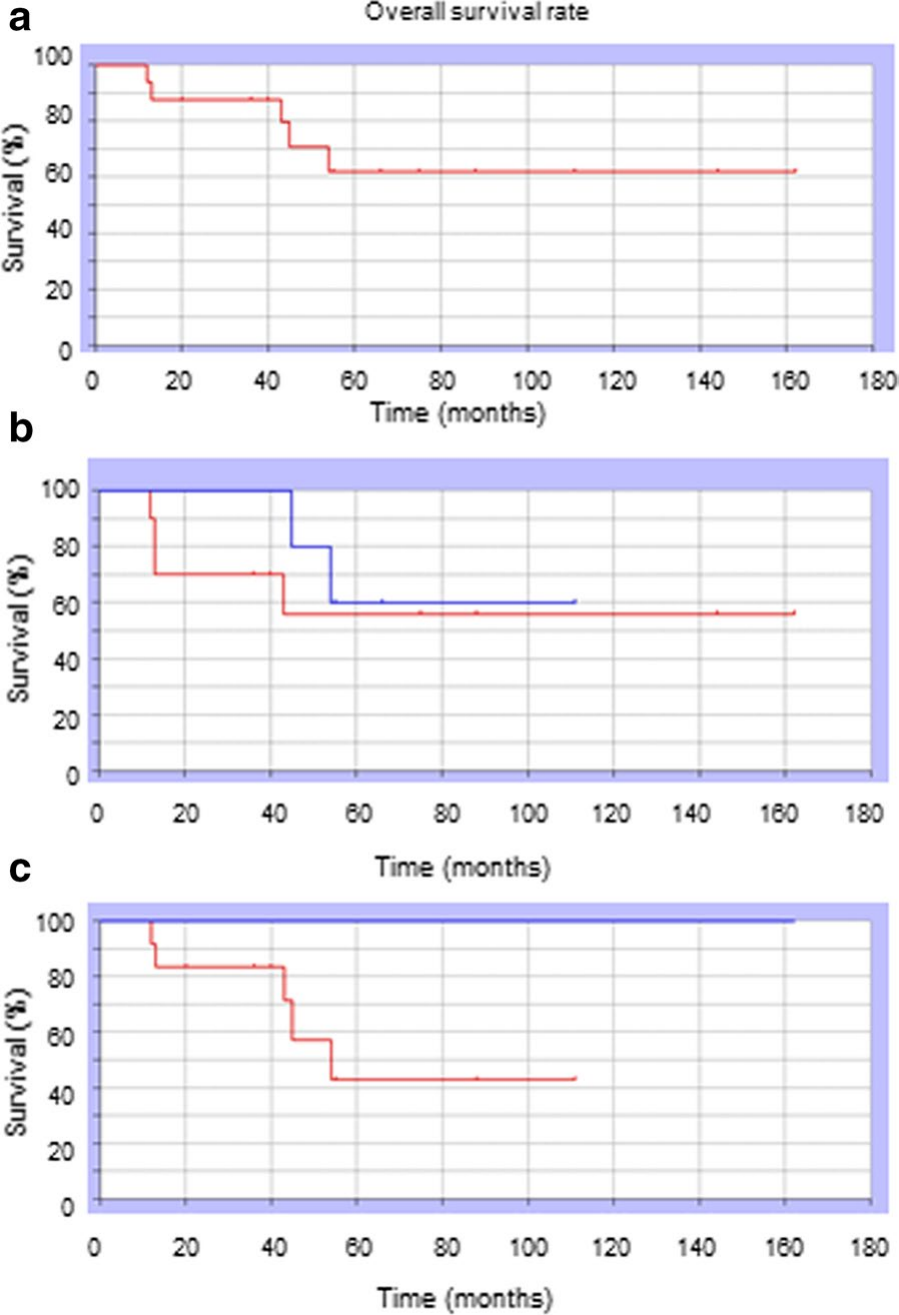
**a** Survival rates of the 18 patients with malignant bone or soft tissue tumors. The Kaplan–Meier method was used to generate survival curves. The 3-year survival rate was 61.36%. **b** Prognosis according to age. The Kaplan–Meier method was used to generate survival curves. The blue line represents the survival of younger patients (< 33 years). The red line represents the survival of older patients (≥ 33 years). The 3-year survival rates of the two groups were 60% and 56%, respectively. There was no significant difference in the 3-year survival rate based on age (P = 0.46). **c** Prognosis according to histological grade. The Kaplan–Meier method was used to generate survival curves. The blue line represents the survival of patients with low-grade sarcomas. The red line represents the survival of patients with high-grade sarcomas. The 3-year survival rates of the two groups were 100% and 42.8%, respectively. There was no significant difference in the 3-year survival rate based on tumor grade (P = 0.08)

We followed up patients for 8–162 (mean: 44) months. Two patients with bone tumors and two with soft tissue tumors developed local recurrence. Two patients with bone tumors and three with soft tissue tumors developed distant metastasis. Among patients with bone tumors, five were continuously disease-free (CDF), one had no evidence of disease (NED), and two were DOD. Among patients with soft tissue tumors, five were CDF, two had NED, and three were DOD. The 3-year survival rate of all of the patients was 61.3% (Fig. 1a). The 3-year survival rate of younger patients (< 33 years) was 56% and that of older patients (> 33 years) was 60%. There was no significant difference in the 3-year survival rate based on age (P = 0.46, Fig. 1b). The 3-year survival rate among patients with high-grade sarcoma was 42.8% and that among patients with low-grade sarcoma was 100%. There was no significant difference in the 3-year survival rate based on sarcoma grade (P = 0.08, Fig. 1c).

We also surveyed the features of the DOD cases. Three of five (60%) patients in the DOD group had ≥ stage IV disease. All patients who had R1 margins developed recurrence. Moreover, all patients with an ASA-PS ≥ 2 died.

### Discussion

It is vital to identify the optimal treatment of sarcomas in AYA patients. However, there is still little evidence, and protocols on how to treat sarcomas in AYA patients have not been clarified in detail. In the current study, we reviewed sarcoma patients treated surgically in our hospital and analyzed which types of patients had poor outcomes. Patients with late-stage sarcomas, lower tumor margin (R1), or a high ASA-PS score had a poor prognosis.

The 5-year survival rate in AYA sarcoma patients is approximately 70% [10, 16], although prognoses in AYA patients may vary depending on age [16]. Our previous study showed that the 5-year survival rate of elderly sarcoma patients was 86% [17]. In the current study, the 3-year survival rate was poorer (61.36%) than those previously reported [17] and that of elderly patients in our hospital. There was also no significant difference in survival rates based on age among these AYA patients. These findings suggest that the AYA generation itself may be a poor prognostic factor.

In general, high-grade sarcoma has a poorer prognosis than that of low-grade sarcoma [18]. Aggressive high-grade malignancies often arise in AYAs [19]. Additionally, late-stage sarcomas have a poorer prognosis than that of early-stage sarcomas [20]. Approximately 80% of AYA sarcoma patients are diagnosed at an early stage [21]. In the current study, we found that AYA sarcoma patients with late-stage sarcomas had a poorer prognosis than that of patients with early-stage sarcomas. These findings suggest that early diagnosis is necessary to obtain a favorable outcome for AYA sarcoma patients.

A Canadian registry showed that the mean operation time for sarcoma was 4 h, and an operation time > 5 h increased the likelihood of reoperation because of wound complications, such as infection [22]. In elderly sarcoma patients, the mean operation time is 114.7 min and the mean blood loss is 160.7 mL [11]. Additionally, a previous study showed that the infection rate after surgical treatment for sarcomas was 23.3% [23]. In the current study, there was no reoperation and no patients developed infection; however, the operation time and intraoperative blood loss were longer and larger, respectively, than those previously reported for elderly sarcoma patients [17].

Achieving a wide margin is important to obtain favorable outcomes [17, 24, 25]. In the current study, all patients with inadequate margins after surgical treatment experienced recurrence. These findings suggest that achieving an R0 ≥ surgical margin is important in the surgical management of AYA sarcoma patients to obtain a favorable prognosis.

The ASA-PS is a general condition classification by the American Society of Anesthesiologists [26]. Recently, Iwai et al. reported that the prognosis of elderly sarcoma patients is correlated with ASA-PS before surgery [27]. In the current study, all patients with an ASA-PS ≥ 2 died. These findings suggest that the ASA-PS may also influence the prognosis or outcome of AYA sarcoma patients undergoing surgical treatment.

## Limitations

Our study had some limitations. First, the number of patients was small. However, the method of statistical analysis is valid. Second, the included tumors were considerably diverse. Furthermore, we were unable to compare the outcomes of these patients to those of younger patients with sarcoma. A future comparative study has been planned to address this point.

In summary, we assessed the clinical features and outcomes of AYA sarcoma patients treated surgically in our hospital. Early detection and appropriate surgical margins are of utmost importance for obtaining a good prognosis in the management of AYA sarcoma patients.

## Data Availability

All data generated or analyzed during this study are included in this published article.

## Abbreviations

AYA: Adolescent and young adult
ECOG: European Society for Medical Oncology Guidelines
ASA-PS: Anesthesiologists-physical status
